# Kaempferol 3-O-Rutinoside, a Flavone Derived from *Tetrastigma hemsleyanum* Diels et Gilg, Reduces Body Temperature through Accelerating the Elimination of IL-6 and TNF-α in a Mouse Fever Model

**DOI:** 10.3390/molecules29071641

**Published:** 2024-04-05

**Authors:** Weilong Zheng, Haina Wang, Xue Wang, Xin Li, Jiahuan Hu, Xiangyu Zi, Yufeng Zhou, Duotao Pan, Yongqian Fu

**Affiliations:** 1 School of Life Science, Taizhou University, No. 1139 Shifu Road, Taizhou 318000, China; z3037713@163.com (W.Z.); wangxueiae@163.com (X.W.); lixin2013@tzc.edu.cn (X.L.); 2 Taizhou Research Institute of Bio-Medical and Chemical Industry Co., Ltd., Taizhou 318000, China; 3 Liaoning Key Laboratory of Hematopoietic Stem Cell Transplantation and Translational Medicine, Department of Hematology, The Second Hospital of Dalian Medical University, Dalian 116027, China; annah_w@foxmail.com; 4 Taizhou Key Laboratory of Biomass Functional Materials Development and Application, Taizhou University, Taizhou 318000, China; hu1366257348@163.com (J.H.); imzixiangyu@163.com (X.Z.); 212748035@njnu.edu.cn (Y.Z.); 5 Institute of Information and Engineering, Shenyang University of Chemical and Technology, Shenyang 110142, China; panduotao@126.com; 6 Institute of Biomass Resources, Taizhou University, Taizhou 318000, China

**Keywords:** fever, traditional Chinese medicine, natural product, cytokines, IL-6

## Abstract

Fever is a serious condition that can lead to various consequences ranging from prolonged illness to death. *Tetrastigma hemsleyanum* Diels et Gilg (*T. hemsleyanum*) has been used for centuries to treat fever, but the specific chemicals responsible for its antipyretic effects are not well understood. This study aimed to isolate and identify the chemicals with antipyretic bioactivity in *T. hemsleyanum* extracts and to provide an explanation for the use of *T. hemsleyanum* as a Chinese herbal medicine for fever treatment. Our results demonstrate that kaempferol 3-rutinoside (K3OR) could be successfully isolated and purified from the roots of *T. hemsleyanum*. Furthermore, K3OR exhibited a significant reduction in rectal temperature in a mouse model of fever. Notably, a 4 μM concentration of K3OR showed more effective antipyretic effects than ibuprofen and acetaminophen. To explore the underlying mechanism, we conducted an RNA sequencing analysis, which revealed that PXN may act as a key regulator in the fever process induced by lipopolysaccharide (LPS). In the mouse model of fever, K3OR significantly promoted the secretion of IL-6 and TNF-α during the early stage in the LPS-treated group. However, during the middle to late stages, K3OR facilitated the elimination of IL-6 and TNF-α in the LPS-treated group. Overall, our study successfully identified the chemicals responsible for the antipyretic bioactivity in *T. hemsleyanum* extracts, and it answered the question as to why *T. hemsleyanum* is used as a traditional Chinese herbal medicine for treating fever. These findings contribute to a better understanding of the therapeutic potential of *T. hemsleyanum* in managing fever, and they provide a basis for further research and development in this field.

## 1. Introduction

Fever is a common symptom associated with various diseases, and if not properly managed, it can lead to serious consequences, including prolonged illness or even death [1,2,3]. While there are several drugs available to reduce high body temperature in adults, many of these drugs are not suitable for children due to the risk of adverse reactions. Commonly used drugs, such as acetaminophen and ibuprofen, which are considered safe for children, have been associated with adverse effects, such as hepatotoxicity, renal toxicity, and complicated infections [4,5,6,7]. Therefore, there is a need to develop more effective and safer therapeutic drugs for fever management in children.

Traditional Chinese medicines (TCMs) have been widely used in East Asia, particularly in China, for thousands of years. Some TCM formulas and individual herbal drugs have been clinically proven to effectively reduce fever [8,9,10]. One such herbal medicine is *Tetrastigma hemsleyanum* Diels et Gilg (*T. hemsleyanum*), which has been used in TCM for centuries [11,12]. Historical records indicate that the roots of *T. hemsleyanum* have been used to treat high fever, pneumonia, and venomous snake or insect bites in children [11,13]. Modern pharmacological studies have also demonstrated various beneficial effects of *T. hemsleyanum*, including anti-inflammatory, antibacterial, antiviral, hypoglycemic, immunomodulatory, and antineoplastic effects [12,14,15,16,17]. *T. hemsleyanum* is often used as an ingredient in Chinese patent medicines for various conditions. However, there is limited research on the constituents of *T. hemsleyanum* that specifically target fever. Given the complex composition of *T. hemsleyanum*, it is crucial to identify the specific chemical(s) responsible for its antipyretic activity. In our study, we comprehensively evaluated the efficacy of *T. hemsleyanum* and its chemical constituents in treating fever.

To assess the antipyretic effect of *T. hemsleyanum*, we established a fever model in mice. We then used various separation methods to isolate the chemicals from *T. hemsleyanum* that significantly reduced body temperature in the mice model. Subsequently, these chemicals were identified using several characterization methods. Additionally, we measured the levels of cytokines associated with fever through the use of ELISA kits (Solarbio, Beijing, China).

## 2. Results

### 2.1. Determination and Analysis of Components of T. hemsleyanum Extracts

**Figure A1:**
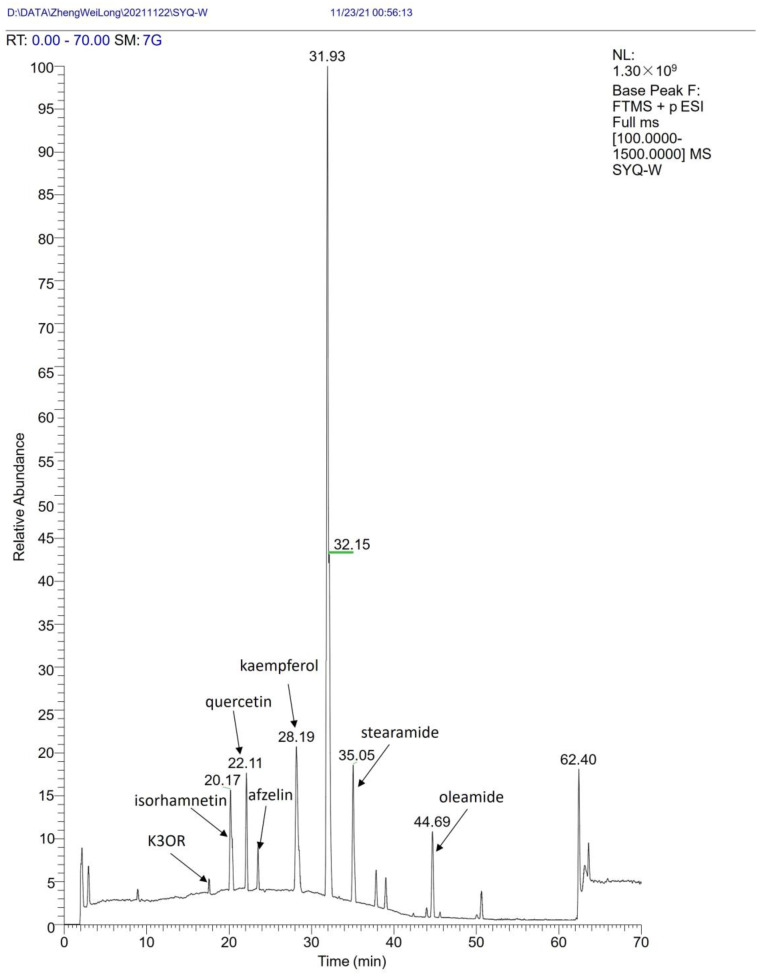
The UHPLC–MS result of *T. hemsleyanum* extracts.

To identify the active components present in the *T. hemsleyanum* extracts, an UHPLC–MS analysis was employed. First, the different chemicals within the *T. hemsleyanum* extracts were separated using a UHPLC system. Subsequently, these separated components were analyzed using MS (Appendix A Figure A1). The obtained results were then compared against various databases, including the natural products mass database (OTCML, ChemSpider, mzCloud, and Arita Lab 6549 Flavonoid Structure Database), for identification purposes. Based on the analysis, the *T. hemsleyanum* extracts were found to contain a variety of constituents, including polysaccharides, phenolic acids, flavonoids, cardiac glycosides, terpenoids, steroids, amino acids, and oil.

### 2.2. T. hemsleyanum Extracts and Components Reduce Rectal Temperature in Fever Model Mice

**Figure 1:**
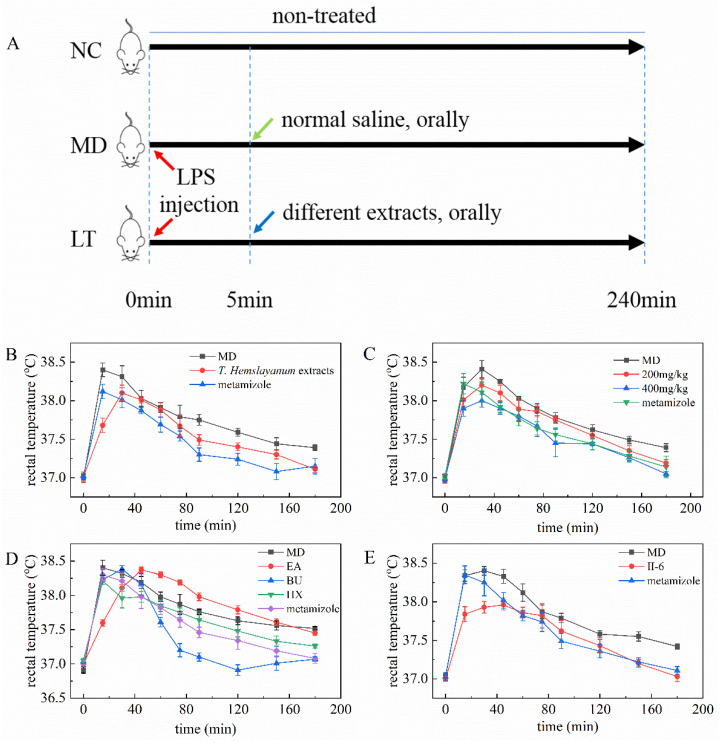
Design of the animal experiment and the effects of *T. hemsleyanum* extracts on fever in the mice model. (**A**) Design of the animal experiment; (**B**) Effects of *T. hemsleyanum* extracts on fever in the mice model; (**C**) Effects of *T. hemsleyanum* extracts with different concentrations on fever in the mice model; (**D**) Effects of *T. hemsleyanum* extracts with different polarity on fever in the mice model; (**E**) Effects of II-6 on fever in the mice model. The rectal temperature of the NC group mice remains at 37 ± 0.3 °C.

**Figure A2:**
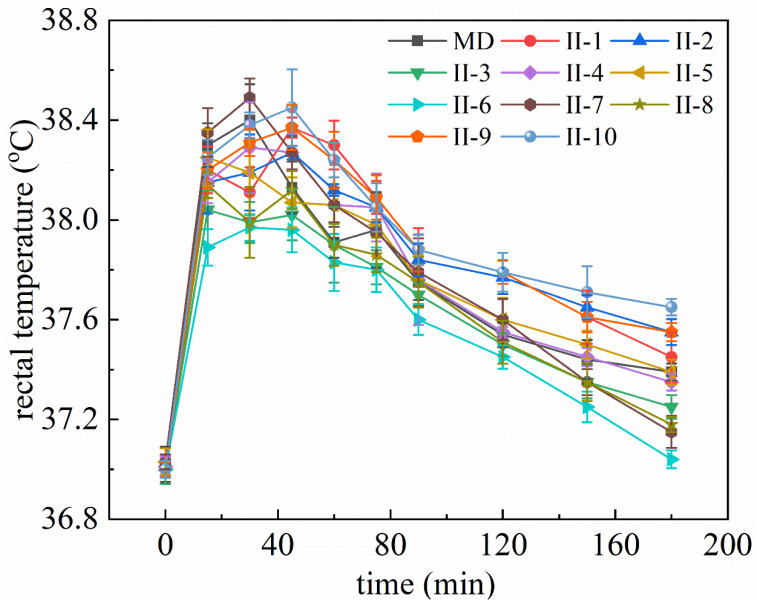
The antipyretic effect of ten chemicals separated from Fraction-II on the fever model mice.

When comparing the LT group to the MD group, a significant reduction in rectal temperature was observed, indicating the potential of *T. hemsleyanum* extracts in reducing fever (Figure 1B). This observation prompted further investigation using 200 and 400 mg/kg concentrations of the *T. hemsleyanum* extracts. The results demonstrate that higher concentrations of the *T. hemsleyanum* extracts yielded a more pronounced reduction in rectal temperature (Figure 1C). Subsequently, four solvents with varying polarity were employed to extract the different ingredients. The activity of the extracted components was then evaluated. Interestingly, the BU extract exhibited a significant reduction in the rectal temperature of the model mice, while the other extracts did not show the same effect, except for the CHL extract, which had insufficient quantity for testing (Figure 1D). Further separation of the BU extract was conducted using silica gel chromatography, resulting in two fractions. Among these fractions, only Fraction II displayed the potential to reduce the rectal temperature of the model mice. Preparative HPLC was subsequently utilized, leading to the isolation of ten chemicals. The activity of these chemicals was assessed, and it was observed that No. 6 (II-6) displayed a substantial reduction in the rectal temperature of the model mice (Figure 1E). It is important to note that the activity of the remaining nine chemicals was lower than that of II-6 (Appendix A Figure A2). These findings collectively suggest that, as the purification level increased, the primary fever-reducing chemical was specifically targeted and isolated.

### 2.3. Structural Characterization of II-6

**Figure A3:**
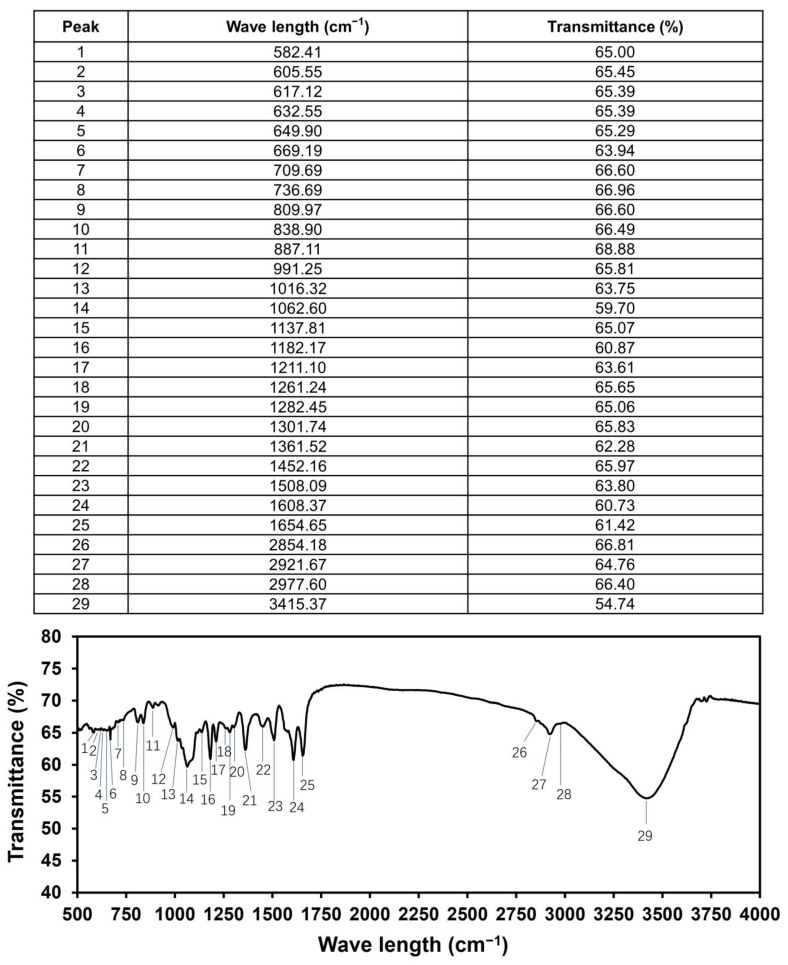
The structure of II-6 was determined by Fourier transform infrared spectroscopy.

**Figure A4:**
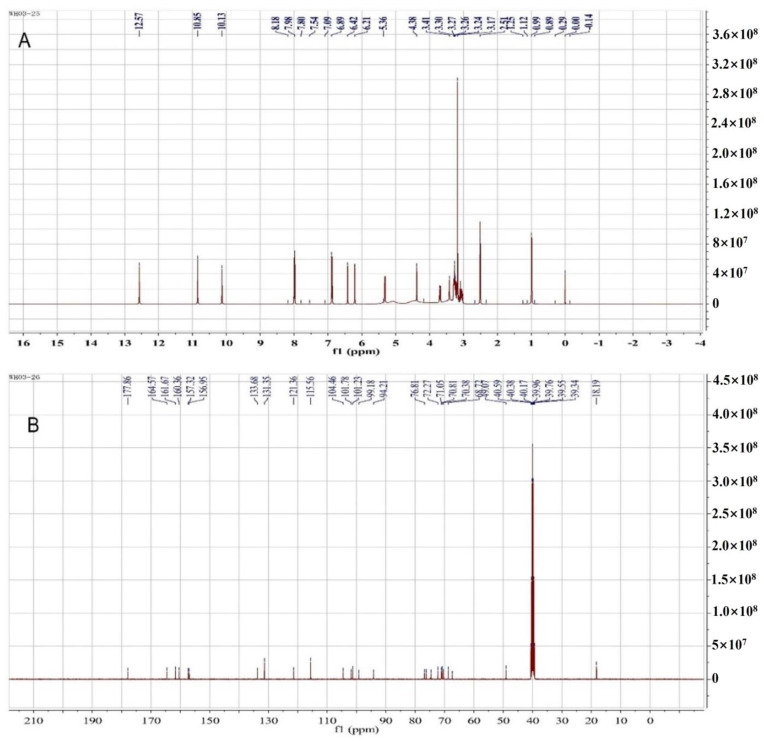
The ^1^H-NMR (**A**) and ^13^C-NMR (**B**) of II-6.

**Figure A5:**
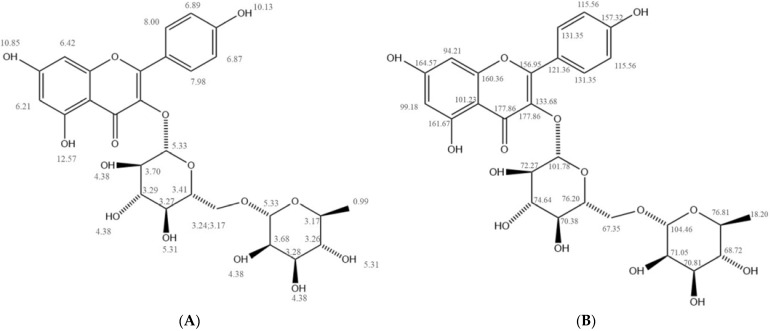
The chemical shift characterized by ^1^H-NMR (**A**) and ^13^C-NMR (**B**).

For the identification of the structure of II-6, initial screening was conducted using FTIR (Appendix A Figure A3). The results revealed the presence of various functional groups, including a hydroxyl group (3415 cm^−1^), methyl group (2977–2854 cm^−1^), aromatic group (1608–1452 cm^−1^), carbonyl group (1654 cm^−1^), and ether group (1211–1062 cm^−1^), among others. Subsequently, ^1^H-NMR and ^13^C-NMR analyses were performed using DMSO-d_6_ as the solvent (Appendix A Figure A4A,B). These analyses aided in characterizing the tentative chemical shift (Appendix A Figure A5A,B), and the obtained data aligned with those of kaempferol 3-rutinoside standards. As a result, it was determined that II-6 could be identified as kaempferol 3-rutinoside (K3OR).

### 2.4. The Effect of Different Concentration of K3OR on the Rectal Temperature of Fever Model Mice

**Figure 2:**
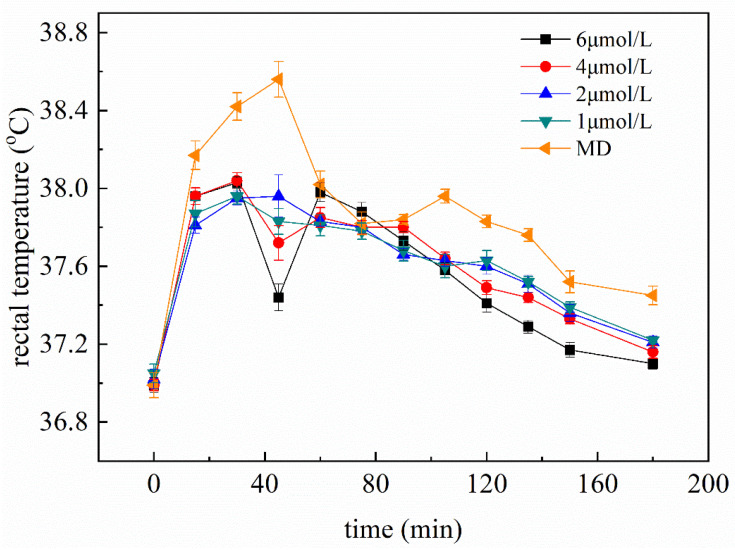
The antipyretic effect of different concentrations of K3OR on the fever model mice. The rectal temperature of the NC group mice remains at 37 ± 0.3 °C.

To assess the antipyretic effect of K3OR, different concentrations of K3OR (1, 2, 4, and 6 μM) were administered to the fever model mice (Figure 2). The results demonstrate that, as the concentration of K3OR increased, the rectal temperature of the fever model mice decreased from 30 min to 180 min. Notably, the 4 and 6 μM groups exhibited superior antipyretic effects compared to the other groups. In particular, in the 6 μM group, the rectal temperature of the fever model mice reduced to a level below 37.3 °C within 150 min. Interestingly, an unexpected decrease in rectal temperature was observed in the 4 and 6 μM groups. One possible explanation for this phenomenon is that K3OR may directly interact with the hypothalamus, thereby lowering the body’s temperature set point and resulting in a decrease in body temperature within 45 min. However, since LPS was still present in the mice model, it is likely that the body temperature increased again, as the LPS had not yet been eliminated.

### 2.5. Comparison of the Effects of K3OR, Ibuprofen, and Acetaminophen

**Figure 3:**
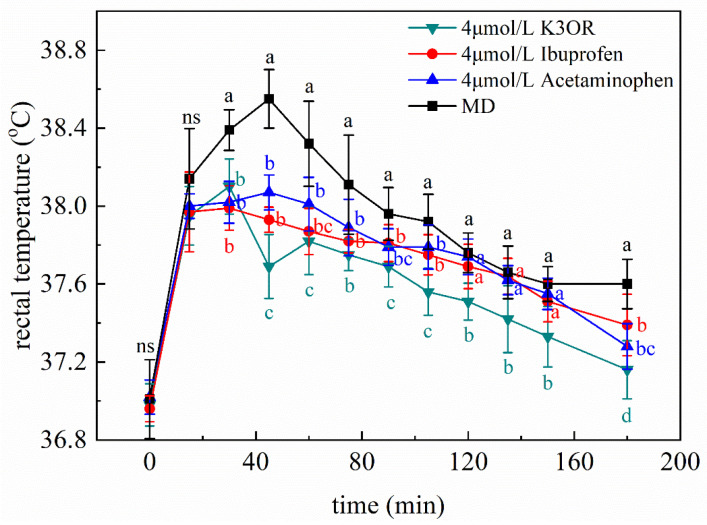
The antipyretic effect of 4 μmol/L K3OR, ibuprofen, and acetaminophen. Different lowercase letters indicate significant differences among different treatments at the same time, whereas ns represents no significance. The rectal temperature of the NC group mice remains at 37 ± 0.3 °C.

To further investigate the effects of K3OR, two commercially available antipyretic drugs, ibuprofen and acetaminophen, were employed to evaluate their influences on the temperature reduction in the fever model mice. The fever model mice were treated separately with 4 μM K3OR, ibuprofen, and acetaminophen (converted to a dose of 9.47 mg/kg, 3.30 mg/kg, and 2.42 mg/kg, respectively). The results indicate that K3OR exhibited a more pronounced reduction in rectal temperature than ibuprofen and acetaminophen. Notably, both ibuprofen and acetaminophen displayed a similar level of antipyretic effects (Figure 3).

### 2.6. Fever-Associated Gene Expression Profiles among NC, MD, and LT Groups

**Figure 4:**
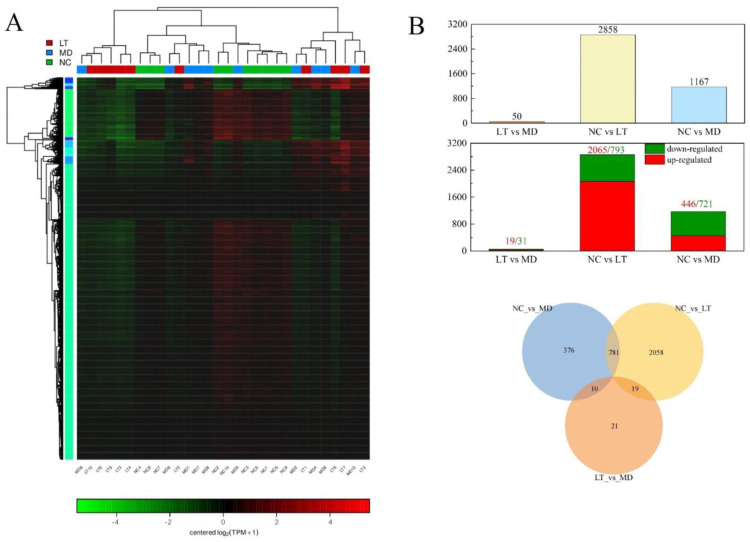
The analysis of differential gene expression. (**A**) Hierarchical clustering. Red represents high expression genes, while green represents low expression genes. The name of the sample was marked as 1 to 30 at the bottom (from left to right in order, MD8, LT10, LT6, LT9, LT3, LT4, NC4, NC8, NC7, MD6, LT5, MD1, MD7, MD5, NC2, NC10, MD3, NC3, NC5, NC1, NC6, NC9, MD2, LT1, MD4, MD9, LT8, LT7, MD10, and LT2, respectively), and the left part is a dendrogram of gene clustering. (**B**) The upper panel presents numbers in three pairwise comparisons, the middle panel presents numbers of up- and downregulated genes in three pairwise comparisons, and the lower panel shows the Venn diagram showing the number of DEGs in the three pairwise comparisons.

**Figure 5:**
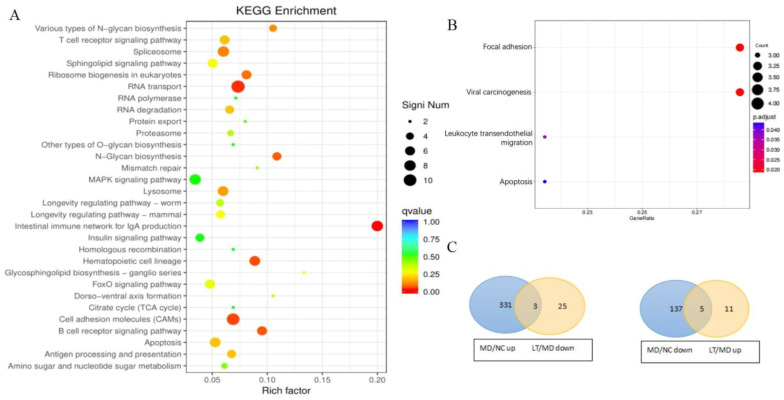
The results of KEGG analyses of the crosstalk genes. (**A**) DEGs and KEGG analyses of the crosstalk genes. (**B**) DEGs and KEGG analyses of the crosstalk genes in LT vs. MD. (**C**) The Venn diagram of genes that are downregulated in LT and upregulated in MD or downregulated in MD and upregulated in LT.

A hierarchical clustering analysis was performed to assess gene expression profiles (Figure 4A). A total of 3339 differentially expressed genes (DEGs) were identified using RESM software (RSEM v1.3.3, qValue < 0.05 and |log_2_FC| > 1) in three pairwise comparisons: NC vs. MD, MD vs. LT, and LT vs. NC. The results are displayed using a Venn diagram (Figure 4B). Subsequently, the DEGs were subjected to the Kyoto Encyclopedia of Genes and Genomes (KEGG) pathway enrichment analysis. Notably, the intestinal immune network for IgA production, N-glycan biosynthesis, and various types of N-glycan biosynthesis were found to be significantly enriched (Figure 5A). Furthermore, compared to the MD and LT groups, focal adhesion, viral carcinogenesis, leukocyte transendothelial migration, and apoptosis pathways were found to be enriched (qValue < 0.05 and |log_2_FC| > 2, Figure 5B). Within these pathways, several genes, including PIK3CD, PXN, XIAP, and ACTN1, displayed significant up- or downregulated expression (Table 1). Moreover, an analysis of the genes involved in the fever pathway and their response to K3OR treatment was conducted. Eight genes were identified, which exhibited downregulation in the LT group and upregulation in the MD group, or vice versa, upon K3OR intervention (Figure 5C and Table 2). Among these genes, only PXN displayed upregulation following LPS treatment and subsequent downregulation after K3OR intervention. Additionally, PXN was found to be involved in the fever pathway, suggesting a potential role as a key regulator in the LPS-induced fever process.

### 2.7. Validation of qRT-PCR and ELISA Assay

**Figure 6:**
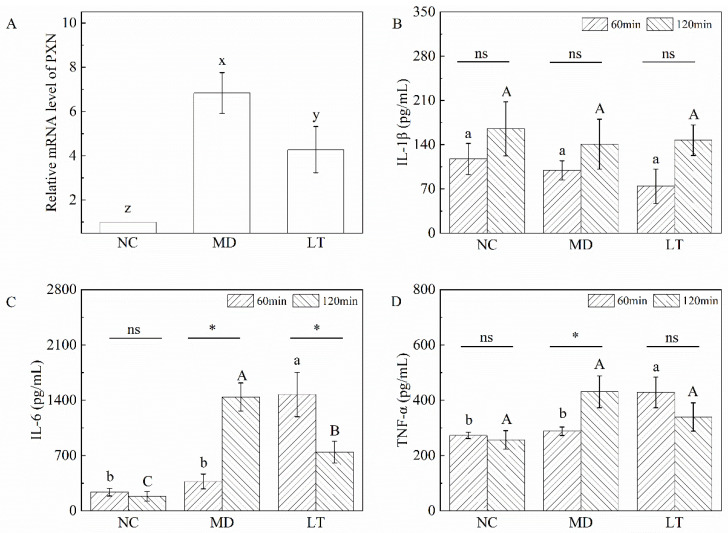
The relative mRNA level of PXN and the level of serum IL-1β, IL-6, and TNF-α in NC, MD, and LT groups. (**A**) Relative PXN mRNA level; (**B**) IL-1β; (**C**) IL-6; (**D**)TNF-α. One-way ANOVA was used to determine the relative mRNA level of PXN and the effects of K3OR on IL-1β, IL-6, and TNF-α at 60 and 120 min, respectively. Paired *t*-test was used to determine the effects of time on IL-1β, IL-6, and TNF-α. x–z represent significant differences among the NC, MD, and LT groups. a, b represent significant differences at 60 min, while A–C represent significant differences at 120 min among the NC, MD, and LT groups. * Represents significant differences between 60 and 120 min in the same group, whereas ns represents no significance.

The expression of PXN was verified via qPCR. The result shows a clear difference between each group: the expression of PXN was upregulated in the MD group compared with that in the NC group (*p* < 0.001), while it was significantly downregulated after K3OR treatment in the LT group (*p* < 0.05, Figure 6A). The results indicate that K3OR treatment can have an antipyretic effect by downregulating PXN expression in the mouse model of LPS-induced fever.

In the presence of LPS, the production of cytokines, such as IL-1β, IL-6, and TNF-α, is triggered, leading to a rise in body temperature. Therefore, the levels of these cytokines in the NC, MD, and LT groups were assessed using an ELISA. The cytotoxic effectors at 60 min revealed that there was no significant change in the level of IL-1β among the three groups. However, the levels of IL-6 and TNF-α displayed a significant difference between the MD and LT groups (Figure 6B–D). These findings suggest that K3OR administration in the LT group led to a rapid stimulation of IL-6 and TNF-α secretion, which could aid in the clearance of LPS in the mouse model. Interestingly, despite the increased levels of IL-6 and TNF-α, the temperature curve demonstrates that high levels of these cytokines did not induce a significant elevation in body temperature. This indicates that K3OR may regulate body temperature by reducing the body’s temperature set point. Similarly, at the 120 min time point, the level of IL-1β remained unchanged among the NC, MD, and LT groups. In contrast, the levels of IL-6 and TNF-α were significantly decreased in the LT group and increased in the MD group. This suggests that the administration of K3OR in the LT group accelerated the elimination of IL-6 and TNF-α compared to the MD group, which is consistent with the temperature curve observed.

## 3. Discussion

Fever is a complex host defense response initiated by pyrogenic activators [18]. The mechanism of fever has been elucidated to a considerable extent, and it can be described as an intentional and active thermoregulatory strategy [19,20]. When the body encounters pyrogenic activators, the defense system, including the innate immune system, is activated. This leads to the recruitment of specific immune cells, which subsequently release cytokines to amplify the immune response. On the one hand, these cytokines activate early non-specific immune responses to recognize pyrogenic activators, such as microbial exogenous pyrogens. On the other hand, during the immune response, cytokines can induce an increase in body temperature. This process is controlled by the thermoregulatory control system located in the anterior hypothalamus/preoptic region [21]. The thermoregulatory control system functions similar to a household thermostat, maintaining a constant body temperature of approximately 37 °C. Fever-related signals can reset the temperature set point to a higher-than-normal value in response to threats until the signals dissipate [22].

Fever is a common symptom that should be given significant attention due to its potential to cause serious consequences, including death. Typically, fever occurs as a result of infectious diseases, such as wounds and trauma. However, it is also reported as a clinical manifestation in collagen vascular diseases and autoimmune diseases. Commercial antipyretic treatments, particularly drugs such as ibuprofen and acetaminophen, have been widely used for decades to reduce fever. However, these drugs have been associated with adverse effects, such as ibuprofen-induced kidney injury, central nervous system depression, epilepsy, and acetaminophen-induced liver injury, particularly in children [6,23,24,25,26]. Therefore, there is an urgent need to explore safer and novel strategies for treating fever.

In our animal experiments, we used LPS and 2,4-dinitrophenol (DNP) to induce fever in mice. LPS induces the secretion of cytokines that, in turn, leads to the occurrence of fever. Conversely, DNP uncouples oxidative phosphorylation by transporting protons through mitochondrial membranes, resulting in the release of large quantities of energy and subsequent fever. However, it was observed that the high temperature induced by DNP dropped rapidly compared to that induced by LPS. The fever induced by LPS could last for more than 3 h, providing an appropriate time window to evaluate the effectiveness of the antipyretic components in our mouse model of fever.

*T. hemsleyanum*, as a traditional Chinese medicine, has been historically proven to have a significant antipyretic effect and a high level of safety. However, unlike Western medicine, the specific mechanism and the bioactive components/chemicals of *T. hemsleyanum* have not been extensively studied. In our study, we collected and identified extracts from *T. hemsleyanum*, which were found to contain flavones, phenolic acids, glycosides, and other compounds. With thousands of chemical candidates, it is challenging to identify the specific antipyretic chemicals directly. Therefore, we used a mouse model of fever along with separation methods, such as reflux extraction, solvent extraction, silica chromatography, and preparative HPLC, to isolate and target the antipyretic chemicals. Each separation method had different efficiencies in eliminating hundreds of chemicals unrelated to antipyretic bioactivity. Finally, one chemical compound was targeted and separated using preparative HPLC. Its structure was identified as kaempferol 3-rutinoside through techniques such as FTIR, ^1^H-NMR, and ^13^C-NMR. After the optimization of the extraction processes, the extraction efficiency of K3OR could reach up to 0.05%.

Based on confirmation through the mouse model of fever, the compound K3OR demonstrated antipyretic bioactivity. K3OR is known to possess various bioactivities, such as anti-adipogenesis, anti-hypertension, anti-α-glucosidase activity, and antitumor [27,28,29,30]. Jang et al. reported that, due to its great α-glucosidase inhibitory ability, K3OR showed an inhibitory effect on adipogenesis to 48.2% without cytotoxicity [27]. K3OR could also play a vasodilatory effect via activation of the cholinergic pathway in rats [28]. Habtemariam found that K3OR is a potent inhibitor of α-glucosidase in vitro with over eight times more activity than acarbose [29]. Compared to anti-adipogenesis, anti-hypertension, and anti-α-glucosidase activity, there is more research focused on the antitumor effect of K3OR. Li et al. found that K3OR could suppress lung adenocarcinoma via the calcium-signaling pathway [30]. In addition, total flavonoids from *T. hemsleyanum*, which mainly contain K3OR, rutinum, isoquercitrin, L-epicatechin, quercetin, astragalin, kaempferol 3-sambubioside, and catechin could inhibit the PI3K/AKT/mTOR pathway and thus delay colorectal tumor growth [31]. The ethanolic extract from seeds of *Euphorbia lathyrism*, which contain abundant K3OR, showed a significant antitumor effect in colon cancer cell lines [32]. However, its antipyretic effect has not been previously reported. K3OR can be isolated from several plant sources, including *Carthamus tinctorius* Linne, *Fagopyrum tataricum* (L.) Gaertn, *Prunus* spp., and *Hosta ventricose* [33,34,35,36]. In our study, K3OR was isolated from *T. hemsleyanum* and showed a novel bioactivity with great potential in treating fever. During the experiment of the mouse model of fever, the temperature fluctuated when the mice orally consumed K3OR, and with increasing doses of K3OR, the fluctuation became more pronounced. One possible explanation for this is that K3OR rapidly adjusts the body’s temperature set point, while LPS continuously induces cytokine secretion, which eliminates the threat at a higher temperature and resets the set point again. We further validated this hypothesis using a mouse model of DNP-induced fever, where body temperature elevation occurs through accelerated ATP oxidation instead of cytokine induction. In this case, the temperature fluctuation disappeared, providing support for the hypothesis. The temperature of the LT group was significantly lower than that of the MD group, indicating the antipyretic effect of K3OR. Furthermore, the temperature in the LT group dropped faster than that in the other two groups. These results collectively demonstrate that K3OR plays an important role in reducing fever in the mouse model of fever.

**Figure A6:**
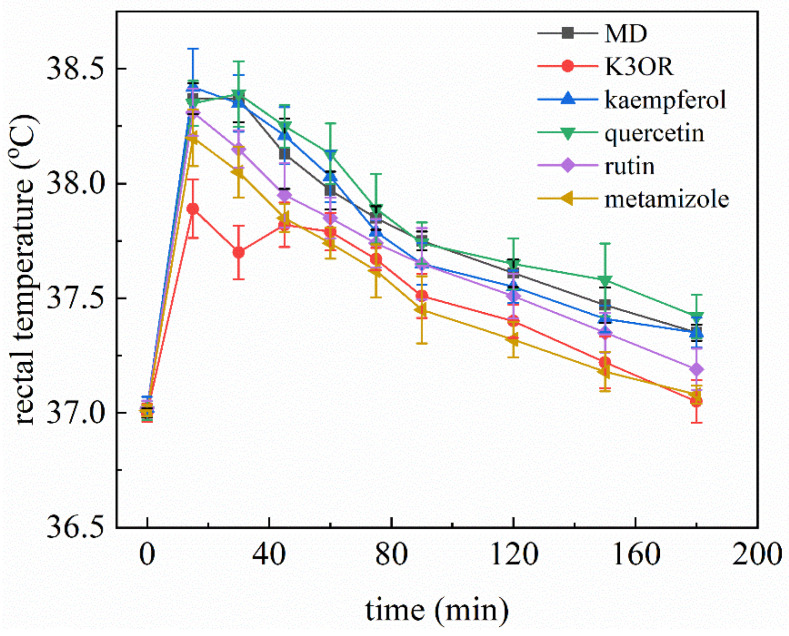
The Comparison of the antipyretic activities of K3OR, kaempferol, quercetin, and rutin.

Flavonoids, as a thoroughly studied class, possess antipyretic activity through diverse mechanisms. In vitro studies have shown that kaempferol (10–50 μM) can reduce the expression of IL-1β and IL-6 in BCRD cells by inhibiting the COX-2/PGE2 signaling pathway while simultaneously increasing the expression of TGF-β, thereby exerting its antipyretic effects [37]. Chalcones, synthesized in the laboratory and tested at concentrations ranging from 2.51 μM to 27.10 μM in vitro, can exert their antipyretic activity by inhibiting COX, PGE2, NO, and NF-κB [38]. Furthermore, other flavonoids, such as flavone, luteolin-7-glucoside, vitexin, isorhamnetin, morin, quercetin, quercitrin, rutin, naringenin, naringin, taxifolin, (−)-epicatechin, procyanidin B1, pelargonidin, daidzein, genistein, and genistin, tested at concentrations ranging from 12.60 μM to 95.9 μM in vitro also exhibit varying degrees of antipyretic activity by inhibiting the COX-2/PGE2 signaling pathway [39]. Based on a UHPLC–MS analysis, the *T. hemsleyanum* extracts were found to contain several well-known flavonoids, including kaempferol, quercetin, and rutin. In order to compare the differences in antipyretic activity, four chemicals, namely K3OR, kaempferol, quercetin, and rutin, were tested at the same concentration (4 μM) with doses of 9.47 mg/kg, 4.56 mg/kg, 4.81 mg/kg, and 9.72 mg/kg, respectively. Metamizole was used as a positive control. The results, depicted in Appendix A Figure A6, demonstrate that the antipyretic activity of the four chemicals ranked from strongest to weakest as follows: K3OR > rutin > kaempferol ≈ quercetin. These findings are consistent with previously published data [39].

Ensuring drug safety is a crucial consideration in medical treatments. Drugs such as ibuprofen and acetaminophen, which have been used for decades, have been associated with adverse effects ranging from vomiting to hepatic and renal toxicity, especially in children. However, the toxicity and safety profiles of K3OR have not yet been validated, and it is essential to explore these aspects as part of its evaluation as a potential drug candidate. The toxicity of *T. hemsleyanum* extracts has been extensively investigated. For instance, Jiang conducted a toxicological evaluation of the decoction of *T. hemsleyanum* root at a dosage of 15 g/person/day, which is commonly used in folk clinical practice. The study results revealed that the oral LD50 for rats and mice exceeded 100 g/kg and 40 g/kg, respectively [40]. Moreover, a 30-day feeding study administering *T. hemsleyanum* root at doses of 6.25, 12.5, and 25.0 g/kg demonstrated no mortality or toxicity, indicating the long-term use of *T. hemsleyanum* root to be safe and non-toxic. Additionally, an acute toxicity test involving the intragastric administration of crude extracts from *T. hemsleyanum* aerial parts established the maximum tolerated dose in mice to be as high as 80.4 g/kg/d, equivalent to 321.6% of the daily dose based on a human body weight of 60 kg. Throughout the 14-day observation period, no adverse reactions, mortality, or abnormal changes in blood and biochemical indices, organ coefficient, or organ pathology were observed [41]. Furthermore, research has shown that the oral toxicity of formula granules of *T. hemsleyanum* aerial parts is minimal with a maximum tolerable dose exceeding 30.4 g/kg/d when administered through gavage, demonstrating their safety and reliability in clinical dosage [42]. To determine the toxicity of K3OR, we conducted thorough searches using various databases, including Web of Science, Google Scholar, PubMed, Science Direct, China National Knowledge Infrastructure (CNKI), and Springer, utilizing both Chinese and English as retrieval languages. However, no reports on the toxicity of K3OR were found. Based on the comprehensive search results, we can conclude that *T. hemsleyanum* extracts (aerial parts and root) as well as K3OR are safe and non-toxic when used within appropriate dosage guidelines.

Paxillin, encoded by PXN, plays a significant role in a cell’s early response to environmental cues, primarily leading to changes in cell shape and the reorganization of the actin cytoskeleton [43]. During the process of LPS-induced fever, Toll-like receptor 4 (TLR4), the receptor for LPS, is upregulated on the cell membrane through paxillin-mediated mechanisms. This upregulation enhances the formation of the signaling complex LPS–LBP–CD14, resulting in LPS signal transduction and the subsequent secretion of cytokines [44]. In this study, a real-time quantitative RT-PCR analysis was used to investigate the differential expression of PXN among the NC, MD, and LT groups. The results revealed that PXN expression was significantly upregulated in the MD group compared to that in the NC group. However, after K3OR treatment in the LT group, PXN expression was significantly downregulated. These findings are consistent with the results obtained from the RNA sequencing analysis, indicating that PXN may function as a potential key regulator in the LPS-induced fever process. Therefore, K3OR downregulates the mRNA level of PXN, leading to the inhibition of actin cytoskeleton reorganization. Consequently, this inhibits the formation of the LPS–LBP–CD14 complex and the secretion of cytokines, thereby inducing a drop in body temperature in the mouse model of fever.

In the process of LPS-induced fever, cytokines, including IL-1β, IL-6, and TNF-α, are known as the main effector molecules [45]. In the mouse model of fever, K3OR significantly promoted the secretion of IL-6 and TNF-α in the LT group during the early stage, which was inconsistent with the initially proposed hypothesis. One possible explanation for this finding is that K3OR, in the early stage, may accelerate the secretion of IL-6 and TNF-α, leading to the elimination of LPS. However, as the experiment progressed to the middle to late stages, the levels of IL-6 and TNF-α in the LT group became significantly lower than those in the MD group, aligning with the observed body temperature curve. In the MD group, there was no significant change in the level of IL-1β, indicating that IL-6 and TNF-α were the main molecules mediating the process of LPS elimination and fever, which is consistent with previous studies [46,47].

As a traditional herbal medicine, *T. hemsleyanum* has been used for centuries without a clear understanding of its specific bioactive chemicals and mechanisms. However, the isolated compound K3OR, obtained from *T. hemsleyanum* extracts, has been validated as the primary bioactive chemical responsible for treating fever. It holds significant potential for further development as a more effective and safer drug for fever reduction.

## 4. Materials and Methods

### 4.1. Preparation of T. hemsleyanum Extracts and Separation of Constituents

*T. hemsleyanum* was sourced from a farm located in Taizhou, Zhejiang, China, at coordinates E 121°30′19.313″ and N 28°23′35.202″. The plant material was authenticated by Dr. Xue Wang, and a voucher specimen has been deposited in our lab (voucher no. T. hemsleyanum-20210110-001). The belowground part of *T. hemsleyanum* was dried and subsequently ground into a fine powder, which was stored at room temperature. Reflux extraction was employed to obtain *T. hemsleyanum* extracts, following the procedure outlined below: A round-bottom flask was used, into which 50 g of *T. hemsleyanum* and 200 mL of 95% ethanol were added. The flask was then connected to a reflux device, and the mixture was subjected to reflux extraction for 1 h at a temperature of 100 °C. After completion of the first extraction, the extracting solution was collected. This extraction process was repeated two more times. Subsequently, the extracting solvent was substituted with 50% ethanol, and the extraction process was carried out three more times. After all extraction steps, the collected solution was combined, and the ethanol in the solvents was removed using a rotary evaporator (Yarong, Shanghai, China). The resulting concentrated solution was then sequentially subjected to extraction with n-hexane, ethyl acetate, chloroform, and n-butanol. Each of these extractions yielded the corresponding extracts, namely HX (n-hexane extract), EA (ethyl acetate extract), CHL (chloroform extract), and BU (n-butanol extract), respectively, after the removal of the solvents.

### 4.2. Animal Experiment

To provide a clearer understanding of the animal experiment, Figure 1A illustrates the design and procedures involved. Male Kunming mice, aged 8 weeks and weighing between 40 and 50 g, were obtained from Zhejiang Vital River Laboratory Animal Technology Co., Ltd. Tongxiang Branch (Jiaxing, China) under permission number SCXK 20210006. Animal husbandry was conducted in accordance with standard laboratory conditions (a temperature of 25 ± 0.5 °C, a relative humidity of 40–70%, 12 h light/dark cycle, and free access to food and water). This study’s protocol adhered to international ethical guidelines and received approval from the Institutional Animal Care and Use Committee of Taizhou University (TZXYBS001).

Prior to the experiments, all mice were acclimatized in an animal room at a temperature of 25 °C for at least 24 h. The rectal temperature of each mouse was then measured using an electronic thermometer (Hynaut, Qingdao, China). Only mice with a thermometer reading of 37 ± 0.3 °C were considered eligible candidates. Subsequently, a fever model was induced by subcutaneously injecting lipopolysaccharide (LPS) at a dose of 20 μg/kg. The mice exhibiting a clearly elevated rectal temperature (above 38 °C) were classified as successfully established fever models.

Initially, the mice without any treatment were allocated to the NC group (n = 10). The remaining model mice were divided into three groups (n = 10 per group): the metamizole group, MD group, and LT group. In the LT group, *T. hemsleyanum* extracts at doses of 200 mg/kg and 400 mg/kg (0.1 mL) were intragastrically administered to the mice. In contrast, the MD group received an equivalent volume of normal saline, and the metamizole group received an equivalent volume of metamizole (5 mg/kg) dissolved in normal saline using the same administration route. Subsequently, the rectal temperature was measured every 15 min until it reduced to 37 °C. The antipyretic effects of the HX (n-hexane), EA (ethyl acetate), CHL (chloroform), and BU (n-butanol) extracts were evaluated using the same protocol with the dose adjusted to 20 mg/kg.

### 4.3. Separation and Purification of Chemicals from BU Extract

Silica gel column chromatography was utilized to separate the various components present in the BU extract. Silica gel with a mesh size of 200–300 was soaked in ethanol overnight and then loaded into a column with a diameter of 3.5 cm and a final gel height of 35 cm. Subsequently, 5.0 g of the BU extract was introduced into the column. Once all the BU extract had permeated through the silica gel, elution was performed using 90% ethanol at a flow rate of 1.5 mL/min. This process resulted in the collection of two fractions, namely Fraction I and Fraction II.

Further separation of Fraction II was carried out using a semi-preparative HPLC system equipped with a YMC preparative column (YMC-Pack ODS-A, 20 × 250 mm, 10 µm) and monitored at 285 nm. The elution conditions were as follows: 0–10 min with a 10% acetonitrile/water mixture, 10.01–40 min with a gradient of acetonitrile/water ranging from 10% to 90%, and 40.01–50 min with 90% acetonitrile/water. After removing the solvents, ten constituents (designated as II-1 to II-10) were obtained. The antipyretic effects of constituents II-1 to II-10 were evaluated using the same protocol with the dose adjusted to 1 mg/kg.

### 4.4. RNA Sequencing Analysis

In accordance with the description in Section 4.2, three groups of mice were treated with the only difference being the administration of kaempferol 3-rutinoside (K3OR) as the drug. Blood samples were collected one hour after intragastric administration. Total RNA was then extracted from the blood samples (10 replicates each for the NC, MD, and LT groups) using the Total RNA Extractor (Trizol) Reagent (Sangon, Shanghai, China) following the manufacturer’s instructions.

An RNA sequencing library was constructed using the Illumina TruSeq^TM^ RNA Sample Prep Kit method (Illumina, San Diego, CA, USA). The concentration and quality of the extracted RNA were evaluated using a Qubit2.0 fluorimeter (Invitrogen, Waltham, MA, USA). Subsequently, the Illumina NovaSeq 6000 platform (LC Science, Houston, TX, USA) was employed for quantification and sequencing following a standard sequencing protocol.

To ensure data quality, the sequencing data were subjected to quality control using Fastx_toolkit_0.0.14, resulting in the generation of clean data. These clean data were then mapped to the reference genome (Rattus_norvegicus, version Rnor_6.0) using HISAT2. Subsequently, the data were assembled using StringTie software (v1.3.3b). Transcript quantification was performed using RSEM (RNA-Seq by expectation-maximization) with the FPKM (fragments per kilobase per million mapped reads) method used to generate read counts. The read counts were subsequently utilized to establish a gene expression profile using DESeq2, applying the default filter conditions (qValue < 0.05 and |log_2_ FC| ≥ 1).

### 4.5. RNA Extraction and Real-Time Quantitative RT-PCR Analysis

In accordance with the description in Section 4.2 and Section 4.4, total RNA was extracted from the blood samples using the Trizol reagent (Invitrogen, Carlsbad, CA, USA), and complementary DNA (cDNA) was synthesized with 1 μg of total RNA by using EasyScript^®^ One-Step gDNA Removal and a cDNA Synthesis Super Mix Kit (TransGen Biotech, Beijing, China). Real-time PCR was performed with an ABI 7500 Fast Real-time PCR System (Applied Biosystems, Carlsbad, CA, USA) and a SYBR Premix Ex Taq^TM^ kit (Takara, Shiga, Japan); β-actin expression was used as a reference. The following temperature profiles were used: initial heating at 95 °C for 30 s, followed by 40 cycles of denaturation at 95 °C for 5 s and annealing and extension at 60 °C for 30 s. The primers used were as follows:

PXN forward: 5′-ACGTCTACAGCTTCCCCAACAA-3′;
PXN reverse: 5′- CTCGATTCGGCTTCATCTGC-3′;
β-actin forward: 5′-GTATCCTGACCCTGAAGTAC-3′;
β-actin reverse: 5′-CCAGAGGCATACAGGGACAG-3′.
Data were analyzed using the 2^−∆∆Ct^ method.

### 4.6. Measurement of Serum Cytokines

The treatment of the three groups of mice followed the description in Section 2.2. Blood samples were collected at both 60 min and 120 min after treatment. These samples were then kept at 4 °C in a refrigerator for 24 h. Afterward, they were centrifuged at 3000 r/min for 10 min at 4 °C. Serum samples were then collected and stored at −80 °C for future use. To measure the levels of cytokines (IL-1, IL-6, and TNF-α), commercial kits (Solarbio, Beijing, China) were employed following the instructions provided by the manufacturer.

### 4.7. Analysis Methods

The molecular weight, chemical group, and composition of II-6 were determined using HPLC–MS, FTIR, and NMR, respectively. The details are as follows:

(1) The molecular weight was determined using UHPLC–MS. HPLC–HRESIMS (Agilent Technologies 1260 Infinity II/6224, Santa Clara, CA, USA) was utilized, and the analysis was conducted on a ZORBAX RRHD chromatography column (2.1 mm × 100 mm, 1.8 µm). The elution condition employed was as follows: 55–90% acetonitrile/water over a period of 0–20 min.
(2) NMR was utilized to determine the chemical composition of the purified samples. Both ^1^H NMR and ^13^C NMR were recorded on a Bruker 400 MHz spectrometer. The samples were dissolved in DMSO-d_6_ for analysis.
(3) FTIR was employed to determine the chemical group of the purified samples. A Fourier transform infrared spectrometer (iS10, Thermo Nicolet, Waltham, MA, USA) was used for data recording. Each sample was mixed with 200 mg of KBr under anhydrous conditions. Subsequently, the mixture was pressed into pellets, which underwent scanning and analysis using the FTIR spectroscope. The spectra were recorded within the frequency range of 4000–400 cm^−1^ at a resolution of 4 cm^−1^.

### 4.8. Statistical Analysis

The results are presented as means ± standard error of the mean (SEM). To assess treatment effects, a one-way ANOVA and a paired T-test were employed. For multiple comparisons, Duncan’s tests were utilized. All statistical analyses were conducted using SPSS software version 22.0 (IBM Corp., Armonk, NY, USA).

## 5. Conclusions

In conclusion, our study represents the first confirmation of the antipyretic effect of kaempferol 3-rutinoside (K3OR) extracted from *T. hemsleyanum*. Our findings demonstrate that K3OR effectively reduces rectal temperature in fever model mice, suggesting its potential as a candidate for the development of an antipyretic drug. Additionally, our study provides insights into the underlying mechanism by which *T. hemsleyanum*, a traditional herbal medicine with a long history of use in China, exhibits antipyretic properties. The collective results of this study provide compelling evidence supporting the promising prospects of K3OR as an effective and safe antipyretic drug for further development.

## Figures and Tables

**Table 1:** KEGG enrichment of LT vs. MD (qValue < 0.05 and |log_2_FC| > 2).

ID	Description	Count	geneID
hsa04510	Focal adhesion	4	PIK3CD/PXN/XIAP/ACTN1
hsa05203	Viral carcinogenesis	4	PIK3CD/PXN/UBR4/ACTN1
hsa04670	Leukocyte transendothelial migration	3	PIK3CD/PXN/ACTN1
hsa04210	Apoptosis	3	PIK3CD/XIAP/CTSD

**Table 2:** Screening of target genes.

Treatment Comparison	Gene Count	Gene Information
MD vs. NC upregulated and LT vs. MD downregulated	3	Bag6, Pxn, Arid5a
MD vs. NC downregulated and LT vs. MD upregulated	5	Wbp1l, Rapgef6, Ifit1bl2, Ice1, Epb41

## Data Availability

Data are contained within the article.

## Abbreviations

TCMs	Traditional Chinese medicines
*T*. *hemsleyanum*	*Tetrastigma hemsleyanum* Diels et Gilg
HPLC	High performance liquid chromatography
UHPLC–MS	Ultra-high performance liquid chromatography–mass spectrometer
NMR	Nuclear magnetic resonance
FTIR	Fourier transform infrared spectroscopy
ELISA	Enzyme-linked immunosorbent assay
HX	n-hexane
EA	Ethyl acetate
CHL	Chloroform
BU	n-butanol
DEGs	Differentially expressed genes
KEGG	Kyoto Encyclopedia of Genes and Genomes
DNP	2,4-dinitrophenol
RSEM	RNA-Seq by expectation-maximization
FPKM	Fragments per kilo base per million mapped reads
LPS	Lipopolysaccharide
